# Bis(1-methyl­piperazine-1,4-diium) tetra­chloridocuprate(II)

**DOI:** 10.1107/S1600536811024354

**Published:** 2011-06-25

**Authors:** Cong-hu Peng

**Affiliations:** a Department of Chemical & Environmental Engineering, Anyang Institute of Technology, Anyang 455000, People’s Republic of China

## Abstract

The title compound, (C_5_H_14_N_2_)[CuCl_4_], was synthesized by hydro­thermal reaction of CuCl_2_ with 1-methyl­piperazine in an HCl/water solution. Both amine N atoms are protonated. The piperazine ring adopts a chair conformation. The Cu—Cl distances in the tetrahedral anion are in the range 2.2360 (7)–2.2732 (7) Å. In the crystal, moderately strong and weak inter­molecular N—H⋯Cl hydrogen bonds link the anion and cation units into an infinite two-dimensional network parallel to the *ab* plane.

## Related literature

For related amino coordination compounds, see: Fu *et al.* (2009 ▶); Aminabhavi *et al.* (1986 ▶); Dai & Fu (2008*a* ▶,*b* ▶). For halogen atoms as hydrogen-bond acceptors, see: Brammer *et al.* (2001 ▶). For the bromide analogue of the title compound, see: Peng (2011 ▶). 
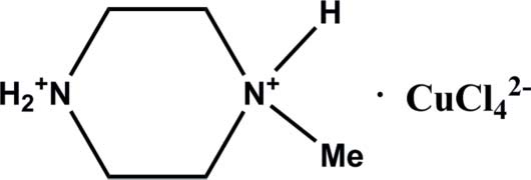


## Experimental

### 

#### Crystal data

(C_5_H_14_N_2_)[CuCl_4_]
*M* *_r_* = 307.52
Orthorhombic, 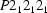

*a* = 8.9717 (18) Å
*b* = 9.945 (2) Å
*c* = 13.753 (3) Å
*V* = 1227.1 (4) Å^3^
*Z* = 4
Mo *K*α radiation
μ = 2.61 mm^−1^
*T* = 298 K
0.20 × 0.05 × 0.05 mm

#### Data collection

Rigaku Mercury2 diffractometer
Absorption correction: multi-scan (*CrystalClear*; Rigaku, 2005 ▶) *T*_min_ = 0.89, *T*_max_ = 1.00
12813 measured reflections
2808 independent reflections
2616 reflections with *I* > 2σ(*I*)
*R*_int_ = 0.036

#### Refinement

*R*[*F* ^2^ > 2σ(*F* ^2^)] = 0.025
*wR*(*F* ^2^) = 0.059
*S* = 1.11
2808 reflections
111 parameters
H-atom parameters constrained
Δρ_max_ = 0.35 e Å^−3^
Δρ_min_ = −0.30 e Å^−3^
Absolute structure: Flack (1983 ▶), 1185 Friedel pairs
Flack parameter: 0.010 (11)

### 

Data collection: *CrystalClear* (Rigaku, 2005 ▶); cell refinement: *CrystalClear*; data reduction: *CrystalClear*; program(s) used to solve structure: *SHELXS97* (Sheldrick, 2008 ▶); program(s) used to refine structure: *SHELXL97* (Sheldrick, 2008 ▶); molecular graphics: *SHELXTL* (Sheldrick, 2008 ▶); software used to prepare material for publication: *SHELXTL*.

## Supplementary Material

Crystal structure: contains datablock(s) I, global. DOI: 10.1107/S1600536811024354/vn2015sup1.cif

Structure factors: contains datablock(s) I. DOI: 10.1107/S1600536811024354/vn2015Isup2.hkl

Additional supplementary materials:  crystallographic information; 3D view; checkCIF report

## Figures and Tables

**Table 1:** Hydrogen-bond geometry (Å, °)

*D*—H⋯*A*	*D*—H	H⋯*A*	*D*⋯*A*	*D*—H⋯*A*
N2—H2*A*⋯Cl3^i^	0.90	2.31	3.179 (2)	162
N2—H2*B*⋯Cl2^ii^	0.90	2.52	3.185 (2)	132
N2—H2*B*⋯Cl1^ii^	0.90	2.65	3.306 (2)	130
N1—H1⋯Cl4	0.90	2.31	3.1895 (19)	164

Symmetry codes: (i) 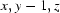; (ii) 
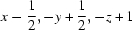
.

**Fig. 1.**
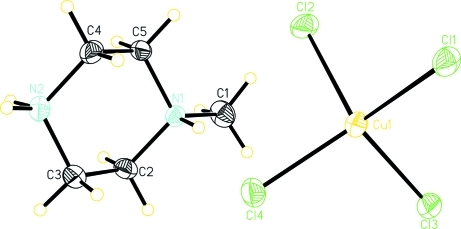
Molecular view of the title compound with the atomic numbering scheme. Displacement ellipsoids are drawn at the 30% probability level.

**Fig. 2.**
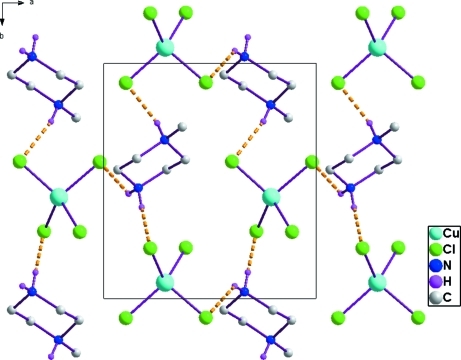
The crystal packing of the title compound viewed along the c axis showing the two-dimensional hydrogen bond network (dashed line). Hydrogen atoms not involved in hydrogen bonding have been omitted for clarity.

## supplementary crystallographic information

### Comment

Amino derivatives of piperazine have found a wide range of applications in material science, due to their magnetic, fluorescent and dielectric properties. There has also been an increased interest in the preparation of amino coordination compounds (Aminabhavi *et al.* 1986; Dai & Fu 2008*a*; Dai & Fu 2008*b*; Fu, *et al.* 2009). We report here the crystal structure of the title compound, *Bis-*(1-methylpiperazine-1,4-diium) tetrachloride copper(II).

The asymmetric unit is composed of one CuCl_4_^2-^ anion, and one 1-methylpiperazine-1,4-diium cation (Fig.1). Both amine N atoms are protonated, thus indicating two positive charges on the 1-methylpiperazine-1,4-diium cation that balance the two negative charges on the CuCl_4_^2-^ anion. The Cu-Cl distances are in the range from 2.2360 (7) to 2.2732 (7) Å, shorter than its bromide analogue in this issue (Peng, 2011). The piperazine ring adopts a chair conformation. The geometric parameters of the title compound are in the normal range.

In the crystal structure, all H atoms of the amine groups are involved in intermolecular N—H···Cl hydrogen bonds with the bond angles ranging from 130.4° to 164.0° and N···Cl distances from 3.179 (2)Å to 3.306 (2)Å, respectively. Following the survey by Brammer *et al.*  (2001), the N2—H2B···Cl1 and N2—H2B···Cl2 H-bonds should be considered to be clearly weaker than the N2—H2A···Cl3 and N1—H1···Cl4 interactions (Table 1). The hydrogen bonds link the cations and anions into an infinite two-dimensional network parallel to the *ab*-plane (Fig.2). The bromide analogue of the title compound is reported elsewhere in this issue (Peng, 2011).

### Experimental

A mixture of 1-methylpiperazine (0.4 mmol), CuCl_2_ (0.4 mmol) and HCl/distilled water (10ml,1:4) sealed in a teflon-lined stainless steel vessel, was maintained at 100 °C. Blue block-shaped crystals suitable for X-ray analysis were obtained after 3 days.

### Refinement

All H atoms attached to C atoms were fixed geometrically and treated as riding on the parent atoms with C-H = 0.97 Å (methylene) and C-H = 0.96 Å (methyl) with *U*_iso_(H) = 1.2*U*_eq_ (methylene) and *U*_iso_(H) = 1.5*U*_eq_ (methyl). The positional parameters of the H atoms (N1, N2) were initially refined freely, subsequently restrained using a distance of 0.90 Å and in the final refinements treated in riding motion on their parent nitrogen atoms with *U_iso_*(H)=1.2*U_eq_*(N).

### Crystal data

**Table:**

(C_5_H_14_N_2_)[CuCl_4_]	*F*(000) = 620
*M**_r_* = 307.52	*D*_x_ = 1.665 Mg m^−^^3^
Orthorhombic, *P*2_1_2_1_2_1_	Mo *K*α radiation, λ = 0.71073 Å
Hall symbol: P 2ac 2ab	Cell parameters from 2808 reflections
*a* = 8.9717 (18) Å	θ = 3.1–27.5°
*b* = 9.945 (2) Å	µ = 2.61 mm^−^^1^
*c* = 13.753 (3) Å	*T* = 298 K
*V* = 1227.1 (4) Å^3^	Block, blue
*Z* = 4	0.20 × 0.05 × 0.05 mm

### Data collection

**Table:**

Rigaku Mercury2 diffractometer	2808 independent reflections
Radiation source: fine-focus sealed tube	2616 reflections with *I* > 2σ(*I*)
graphite	*R*_int_ = 0.036
Detector resolution: 13.6612 pixels mm^-1^	θ_max_ = 27.5°, θ_min_ = 3.1°
profile data from φ scans	*h* = −11→11
Absorption correction: multi-scan (*CrystalClear*; Rigaku, 2005)	*k* = −12→12
*T*_min_ = 0.89, *T*_max_ = 1.00	*l* = −17→17
12813 measured reflections	

### Refinement

**Table:**

Refinement on *F*^2^	Hydrogen site location: inferred from neighbouring sites
Least-squares matrix: full	H-atom parameters constrained
*R*[*F*^2^ > 2σ(*F*^2^)] = 0.025	*w* = 1/[σ^2^(*F*_o_^2^) + (0.022*P*)^2^ + 0.1621*P*] where *P* = (*F*_o_^2^ + 2*F*_c_^2^)/3
*wR*(*F*^2^) = 0.059	(Δ/σ)_max_ < 0.001
*S* = 1.11	Δρ_max_ = 0.35 e Å^−^^3^
2808 reflections	Δρ_min_ = −0.30 e Å^−^^3^
111 parameters	Extinction correction: *SHELXL97* (Sheldrick, 2008), Fc^*^=kFc[1+0.001xFc^2^λ^3^/sin(2θ)]^-1/4^
0 restraints	Extinction coefficient: 0.0271 (9)
Primary atom site location: structure-invariant direct methods	Absolute structure: Flack (1983), 1185 Friedel pairs
Secondary atom site location: difference Fourier map	Flack parameter: 0.010 (11)

### Special details

**Table:**

Geometry. All esds (except the esd in the dihedral angle between two l.s. planes) are estimated using the full covariance matrix. The cell esds are taken into account individually in the estimation of esds in distances, angles and torsion angles; correlations between esds in cell parameters are only used when they are defined by crystal symmetry. An approximate (isotropic) treatment of cell esds is used for estimating esds involving l.s. planes.
Refinement. Refinement of F^2^ against ALL reflections. The weighted R-factor wR and goodness of fit S are based on F^2^, conventional R-factors R are based on F, with F set to zero for negative F^2^. The threshold expression of F^2^ > 2sigma(F^2^) is used only for calculating R-factors(gt) etc. and is not relevant to the choice of reflections for refinement. R-factors based on F^2^ are statistically about twice as large as those based on F, and R- factors based on ALL data will be even larger.

### Fractional atomic coordinates and isotropic or equivalent isotropic displacement parameters (Å^2^)

**Table:**

	*x*	*y*	*z*	*U*_iso_*/*U*_eq_	
Cu1	0.78864 (3)	0.56811 (3)	0.60180 (2)	0.03632 (10)	
Cl2	0.98061 (8)	0.41919 (7)	0.59536 (5)	0.05205 (19)	
Cl3	0.71852 (8)	0.71379 (6)	0.71940 (5)	0.04349 (16)	
N2	0.6621 (2)	−0.0291 (2)	0.58707 (15)	0.0377 (5)	
H2A	0.6784	−0.1116	0.6118	0.045*	
H2B	0.6166	−0.0538	0.5316	0.045*	
Cl4	0.60364 (7)	0.41742 (6)	0.61497 (6)	0.04956 (18)	
N1	0.7864 (2)	0.17565 (17)	0.70981 (13)	0.0304 (4)	
H1	0.7517	0.2466	0.6761	0.037*	
Cl1	0.88660 (8)	0.71628 (6)	0.49755 (5)	0.04457 (17)	
C4	0.7962 (3)	0.0489 (2)	0.55755 (18)	0.0401 (5)	
H4A	0.7658	0.1277	0.5211	0.048*	
H4B	0.8585	−0.0060	0.5158	0.048*	
C5	0.8834 (3)	0.0914 (2)	0.64550 (18)	0.0361 (5)	
H5A	0.9174	0.0126	0.6808	0.043*	
H5B	0.9702	0.1426	0.6256	0.043*	
C1	0.8693 (3)	0.2304 (3)	0.79610 (19)	0.0491 (7)	
H1A	0.8080	0.2948	0.8292	0.074*	
H1B	0.9594	0.2731	0.7746	0.074*	
H1C	0.8935	0.1581	0.8396	0.074*	
C2	0.6547 (3)	0.0954 (3)	0.74224 (18)	0.0383 (6)	
H2C	0.5919	0.1502	0.7838	0.046*	
H2D	0.6882	0.0184	0.7796	0.046*	
C3	0.5663 (3)	0.0481 (3)	0.65572 (18)	0.0397 (6)	
H3A	0.4848	−0.0083	0.6776	0.048*	
H3B	0.5241	0.1252	0.6224	0.048*	

### Atomic displacement parameters (Å^2^)

**Table:**

	*U*^11^	*U*^22^	*U*^33^	*U*^12^	*U*^13^	*U*^23^
Cu1	0.03892 (17)	0.02711 (15)	0.04294 (17)	0.00232 (13)	0.00213 (13)	0.00148 (13)
Cl2	0.0549 (4)	0.0356 (3)	0.0656 (4)	0.0155 (3)	0.0214 (3)	0.0148 (4)
Cl3	0.0472 (3)	0.0367 (3)	0.0466 (3)	−0.0003 (3)	0.0106 (3)	−0.0032 (3)
N2	0.0397 (11)	0.0306 (10)	0.0428 (12)	−0.0029 (8)	−0.0008 (9)	−0.0038 (9)
Cl4	0.0380 (3)	0.0328 (3)	0.0779 (5)	−0.0016 (3)	−0.0067 (3)	0.0037 (3)
N1	0.0304 (9)	0.0270 (9)	0.0340 (10)	0.0017 (8)	−0.0019 (9)	−0.0003 (8)
Cl1	0.0558 (4)	0.0348 (3)	0.0431 (3)	0.0093 (3)	0.0080 (3)	0.0093 (3)
C4	0.0483 (14)	0.0315 (12)	0.0403 (13)	−0.0010 (12)	0.0147 (11)	−0.0016 (11)
C5	0.0288 (12)	0.0280 (12)	0.0516 (14)	0.0007 (10)	0.0079 (10)	0.0019 (11)
C1	0.0456 (15)	0.0592 (17)	0.0424 (14)	−0.0092 (13)	−0.0093 (12)	−0.0046 (13)
C2	0.0324 (13)	0.0435 (14)	0.0391 (13)	−0.0042 (10)	0.0070 (10)	−0.0004 (11)
C3	0.0316 (12)	0.0422 (14)	0.0454 (14)	−0.0038 (11)	−0.0004 (10)	−0.0057 (13)

### Geometric parameters (Å, °)

**Table:**

Cu1—Cl1	2.2360 (7)	C4—H4A	0.9700
Cu1—Cl4	2.2435 (8)	C4—H4B	0.9700
Cu1—Cl3	2.2606 (7)	C5—H5A	0.9700
Cu1—Cl2	2.2732 (7)	C5—H5B	0.9700
N2—C4	1.488 (3)	C1—H1A	0.9600
N2—C3	1.490 (3)	C1—H1B	0.9600
N2—H2A	0.9001	C1—H1C	0.9600
N2—H2B	0.8999	C2—C3	1.505 (3)
N1—C2	1.494 (3)	C2—H2C	0.9700
N1—C5	1.497 (3)	C2—H2D	0.9700
N1—C1	1.502 (3)	C3—H3A	0.9700
N1—H1	0.8998	C3—H3B	0.9700
C4—C5	1.502 (4)		
			
Cl1—Cu1—Cl4	141.52 (3)	N1—C5—C4	109.30 (19)
Cl1—Cu1—Cl3	98.38 (3)	N1—C5—H5A	109.8
Cl4—Cu1—Cl3	99.47 (3)	C4—C5—H5A	109.8
Cl1—Cu1—Cl2	96.12 (3)	N1—C5—H5B	109.8
Cl4—Cu1—Cl2	97.38 (3)	C4—C5—H5B	109.8
Cl3—Cu1—Cl2	131.02 (3)	H5A—C5—H5B	108.3
C4—N2—C3	111.74 (19)	N1—C1—H1A	109.5
C4—N2—H2A	116.6	N1—C1—H1B	109.5
C3—N2—H2A	108.9	H1A—C1—H1B	109.5
C4—N2—H2B	106.1	N1—C1—H1C	109.5
C3—N2—H2B	114.6	H1A—C1—H1C	109.5
H2A—N2—H2B	98.4	H1B—C1—H1C	109.5
C2—N1—C5	109.69 (17)	N1—C2—C3	110.34 (19)
C2—N1—C1	110.44 (19)	N1—C2—H2C	109.6
C5—N1—C1	112.44 (19)	C3—C2—H2C	109.6
C2—N1—H1	107.4	N1—C2—H2D	109.6
C5—N1—H1	109.6	C3—C2—H2D	109.6
C1—N1—H1	107.1	H2C—C2—H2D	108.1
N2—C4—C5	110.40 (19)	N2—C3—C2	110.97 (19)
N2—C4—H4A	109.6	N2—C3—H3A	109.4
C5—C4—H4A	109.6	C2—C3—H3A	109.4
N2—C4—H4B	109.6	N2—C3—H3B	109.4
C5—C4—H4B	109.6	C2—C3—H3B	109.4
H4A—C4—H4B	108.1	H3A—C3—H3B	108.0

### Hydrogen-bond geometry (Å, °)

**Table:**

*D*—H···*A*	*D*—H	H···*A*	*D*···*A*	*D*—H···*A*
N2—H2A···Cl3^i^	0.90	2.31	3.179 (2)	162
N2—H2B···Cl2^ii^	0.90	2.52	3.185 (2)	132
N2—H2B···Cl1^ii^	0.90	2.65	3.306 (2)	130
N1—H1···Cl4	0.90	2.31	3.1895 (19)	164

Symmetry codes: (i) *x*, *y*−1, *z*; (ii) *x*−1/2, −*y*+1/2, −*z*+1.
